# Application of Deep Learning Workflow for Autonomous Grain Size Analysis

**DOI:** 10.3390/molecules27154826

**Published:** 2022-07-28

**Authors:** Alexandre Bordas, Jingchao Zhang, Juan C. Nino

**Affiliations:** 1 Department of Materials Science and Engineering, University of Florida, Gainesville, FL 32612, USA; abordas@ufl.edu; 2 NVIDIA AI Technology Center (NVAITC), Santa Clara, CA 95051, USA

**Keywords:** deep learning, convolutional neural network, pre-trained model, edge detection, grain size

## Abstract

Traditional grain size determination in materials characterization involves microscopy images and a laborious process requiring significant manual input and human expertise. In recent years, the development of computer vision (CV) has provided an alternative approach to microstructural characterization with preliminary implementations greatly simplifying the grain size determination process. Here, an end-to-end workflow to measure grain size in microscopy images without any manual input is presented. Following the ASTM standards for grain size determination, results from the line intercept (Heyn’s method) and planimetric (Saltykov’s method) approaches are used as the baseline. A pre-trained holistically nested edge detection (HED) model is used for CV-based edge detection, and the results are further compared to the classic Canny edge detection method. Post-processing was performed using open-source image processing packages to extract the grain size. In optical microscope images, the pre-trained HED model achieves much higher accuracy than the Canny edge detection method while reducing the image processing time by one to two orders of magnitude compared to traditional methods. The effects of morphological operations on the predicted grain size accuracy are also explored. Overall, the proposed end-to-end convolutional neural network (CNN)-based workflow can significantly reduce the processing time while maintaining the same accuracy as the traditional manual method.

## 1. Introduction

With a global increase in computational resources and refined artificial intelligence (AI) architectures, machine learning (ML) has become a standard methodology for answering increasingly difficult scientific questions. In recent years, convolutional neural networks (CNN) have dominated the image segmentation space by reducing the number of parameters and thus computing time and power when compared to that of a fully connected neural network [1,2]. Similar to multiple growing fields, materials science and engineering have benefitted from the application of CNNs in property prediction [3,4,5], the homogenization of heterogeneous materials [6], and various uses in transmission electron microscopy [7,8,9]. Surprisingly, although there have been several machine learning and semi-automated approaches applied in microstructural characterization (i.e., metallographic or ceramographic analysis) [10,11,12,13,14,15,16,17,18,19,20,21,22,23,24], the use of CNNs in grain-size determination, while growing, has been comparatively minimal.

It is well known that the mechanical, electrical, magnetic, and optical properties of ceramics, metals, and their alloys can all be tailored and modified by changes in microstructures [25]. Consequently, grain size engineering is a common practice used to optimize the physical properties of a material without altering its chemistry [26]. Besides the well-known Hall–Petch equation relating the grain size and mechanical properties of alloys [27], it has been shown, for example, that an increase in the triple junction volume fraction over the grain boundary volume fraction occurs below a critical grain size and causes softening in nanocrystalline materials [28]. In solar cells, photovoltaic parameters improve as the perovskite grain size increases [29]. In heterogeneous materials, the viscoplastic strain rate of grain depends on the size of that specific grain [30]. It is clear that grain size is critical to the characterization and development of new materials.

**Figure 1:**
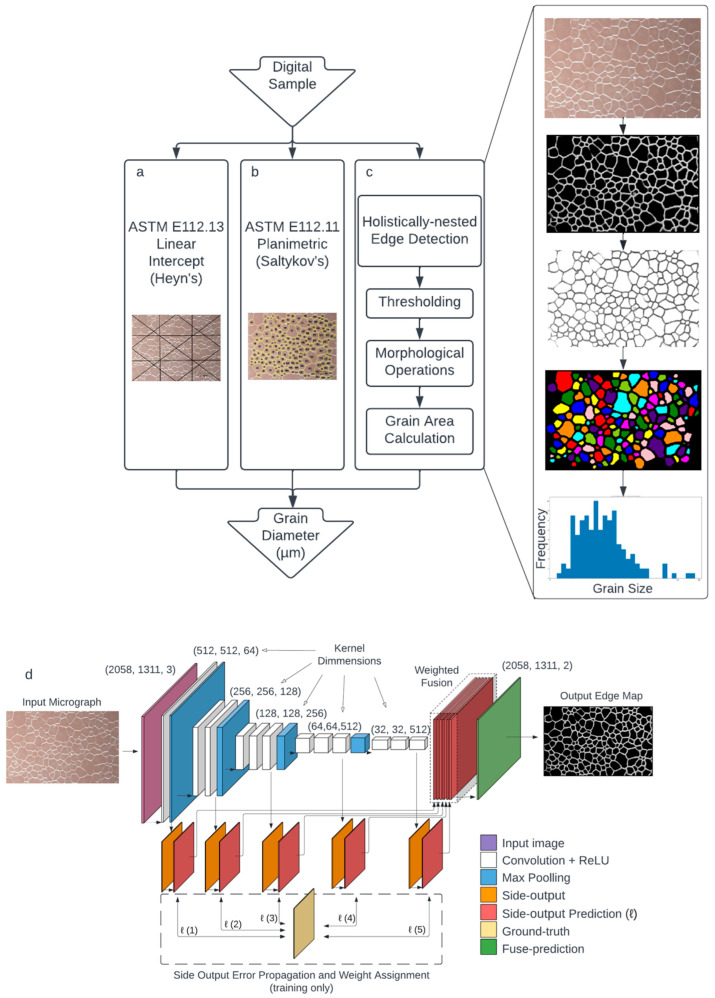
Workflow for methods of determining mean grain diameter from an optical micrograph, including Heyn’s (**a**), Saltykov’s (**b**), and the newly proposed method (**c**). The HED architecture (**d**) follows a VGGNet-inspired convolution and pooling structure but extracts the side output of each last convolutional layer to which predictions are made and compared to the ground truth image. The model composes five stages with strides 1, 2, 4, 8, and 16, each with its own side output. Weights are then assigned to each side-output prediction and combined into a final loss function to create a fuse prediction. The finalized output is a grayscale edge-prediction map with the same height and width as the original image. The intensity of each pixel is associated with the probability that the pixel represents an edge.

Grain size is typically determined via standards such as the one by the American Society for Testing and Materials (ASTM E112-13). Traditionally, an optical microscope or scanning electron microscope (SEM) is used to collect micrographs, which are analyzed using a linear intercept (Figure 1a) or planimetric method (Figure 1b). Normally, when the image contrast between grains and grain boundaries is not evident, the sample is mechanically polished to produce an optically flat, scratch-free surface, and then chemically or thermally treated to preferentially etch the amorphous regions, thus enhancing the grain boundaries when observed under a microscope. Manual grain size determination through line-intercept and planimetric methods is tedious and time-consuming but represents an essential materials characterization quantifier.

It is important to note that the automation of this quantification has been attempted numerous times with a wide range of methods. At its core, automated grain size determination is primarily an issue of edge detection. Image processing techniques such as Canny edge detection facilitate grain size determination by converting original images to masked outlines of grain borders [16,31]. Polarized micrographs processed via Lazy Grain Boundary have produced grain boundary maps of steel microstructures [15]. Peregrina-Barreto et al. used an advanced thresholding technique to discern grains and a planimetric grain count to determine the ASTM grain size, *G* [10]. Past research has also employed Canny edge detection to create closed contours and similarly determine planimetric grain size, with small improvements in accuracy [12]. Zhang-Suen’s thinning algorithm has been shown to reduce processed micrographs to their main edge components [11,32]. Despite these advancements, all these methods have some key drawbacks. Primarily, post-processing manual input is often still needed to connect grain boundaries. Current methods work especially well with some materials but have not been applied to diverse sets of micrographs. In particular, the main focus has been placed on metals samples with little consideration for ceramics.

More recently, AI has been implemented in grain size analysis using trained ML models and CNNs that rely on the availability of large datasets (which are rarely available in a typical laboratory) for training [13,14]. The efficacy of neural network models depends greatly on the amount of data available for training and testing, even in transfer learning [33]. Therefore, using pretrained models that can directly apply complex computer vision algorithms without the input of any additional training data is greatly advantageous. U-Net, for instance, is a deep learning model widely used in medical image segmentation for the detection of various anatomical phenomena [34,35]. These existing pretrained high-accuracy edge detection algorithms have the potential to impact areas outside of their original field of use. Guided by this idea, this paper explores a method of average grain diameter determination using the pretrained holistically nested edge detection algorithm [36] (Figure 1c). This method aims to use the sophistication and universality of a model trained on non-micrograph data to process and characterize the grain size of various materials in an automated fashion.

## 2. Materials and Methods

### 2.1. Sample Preparation and Imaging

Image samples were chosen to have distinct grains entirely in focus. Ceria pellet samples from previously published work were used [37]. Briefly, the samples were synthesized from 99.9% cerium oxide powder sieved to 212 µm particles. After pressing, the pellets were sintered at 1650 °C for 2 h in the air before being annealed at 1750 °C for 10 h to induce grain growth and grain boundary differentiation. Images of the resulting microstructure of 8 mm sample pellets were collected using a Nikon Eclipse LV100 optical microscope with a Lumenera Infinity 1 microscope camera attachment. Images were collected as 1280 by 1024 resolution TIF files, each approximately 9 megabytes in size. Lumenera’s Infinity Analyze software was used to calibrate the scale of the image and to place a scale bar in each bottom-right corner. Five fields (images) were acquired for five samples at a magnification of 10×. The test area for each image was approximately 0.655 mm^2^.

### 2.2. Image Preprocessing

Image preprocessing for grain size analysis typically involves filtering, where Gaussian blur has been used to reduce noise, and local Laplacian filtering to accentuate the presence of edges [11,12]. It is important to note that while HED (described below) performed well without smoothening or an increase in contrast in the tested samples, image filters are likely to be needed in cases with less-well-defined grain boundaries. Moreover, the large TIF files were algorithmically converted to JPG images and cropped to remove the scale bar from the image analysis. If the scale bar is not removed, the HED detects its edges to be a portion of the grain boundary, which is inconsistent with the material sample’s grains. A MATLAB script was used to crop the images to the region above the scale bar: 2058 × 1311 pixels. Nonetheless, to preserve the scale (magnification) information of the image, the pixel-to-micrometer ratio, which is variable from sample to sample, was added to the image file as metadata. In this work, ImageJ [19] and a Jupyter notebook are used to determine the pixel-to-micrometer ratio and store it as Exif metadata under “User Comment”.

### 2.3. Holistically Nested Edge Detection

Edge detection is a fundamental and important topic in computer vision. Various approaches have been proposed to tackle this problem, such as the Canny detector [31], Statistical Edges [38], Structured Edges [39], and DeepEdge [40]. This work used a pretrained holistically nested edge detection (HED) [36] model to extract the grain boundaries in composite microscopy images. Briefly, fully convolutional neural networks inspire the model with trimmed VGG nets for deep supervised learning. The holistic model can make image-to-image predictions that can be used as image edge detectors. The nested keyword refers to the neural network structure, which uses refine-edge maps as side outputs (Figure 1d). This model can learn the hierarchical features, which are crucial in detecting natural image edges and object boundaries. The HED model significantly advances the optimal dataset scale ODS F-scores of the Berkeley Segmentation Dataset and Benchmark (BSD500) [41] and the NYU Depth dataset [42] benchmarks, with an astonishing speed of 0.4 s per image. In this work, the pretrained model on BSD500, which has 200 training, 100 validation, and 200 testing images, is used [41].

### 2.4. Morphological Operations

Morphological operands remove noise while enlarging or shrinking the background of an image [43]. Erosion preserves the foreground pixels only when a structural element entirely matches that of the pixels it covers. The effect is the thinning of foreground images. Dilation works similarly by comparing the structural element to the background, resulting in a thickening of foreground images. Two inputs are needed: Dimensions of the kernel and a binary image. The process is iterated until the desired effect is achieved.

**Figure 2:**
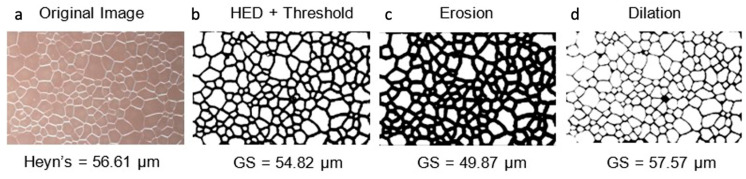
Optical micrograph (**a**) after HED and initial thresholding (**b**), 8 iterations of erosion (**c**), and 8 iterations of dilation (**d**). The values underneath indicate the measured grain size (GS) of the contours using the mean grain diameter determination method outlined below.

In grain size determination, morphological operations alleviate some of the issues encountered when employing an edge detection methodology to a material microstructure. The characteristic effects of erosion and dilation on an optical image is depicted in Figure 2. The HED occasionally interprets imperfect grain boundaries as broken edges, double edges on thick boundaries, or undesired particles in noise locations (Figure 2b). In morphological erosion, broken edges are united and double edges are joined together by a disk structural element, given the distance between interpreted edges is smaller than the distance covered by the kernels. The resulting image contains large grain boundaries and miscellaneous bits, which is misrepresentative of the material sample (Figure 2c). To minimize this artifact, morphological dilation reduces the edge thickness and size of unwanted particles to yield an image much more indicative of the grains (Figure 2d). Achieving a thin closed contour is necessary as the area of each cell determines the grain size. Morphological operations execute this by enlarging and shrinking the grain boundary.

### 2.5. Conventional Grain Size Determination

The ASTM designation E112-13 was used to benchmark the quantitative data from the newly proposed algorithm. Both the line intercept (Heyn’s) and planimetric (Saltykov’s) methods were used as described in the ASTM standard. In the line intercept method, a set of 10 randomly drawn straight lines was placed over the microscopy images, cumulatively spanning the entirety of the image height and width. The intercepts, including all exceptions found in E112-13.13.1, were noted along with line length and converted to micrometers. The pixel-to-micrometer ratio was derived by dividing the length (in pixels) of the scale bar by its labeled value. Intercepts were determined manually by visual inspection, while ImageJ software was used to determine individual line lengths. The total line length divided by total intercepts gave an average grain diameter (length). All samples exhibited relatively equiaxed grains and thus no further specially oriented test lines were needed. 

For the planimetric method, the entire rectangular area of the image is used. Each grain within the image is counted, $N_{inside}$, with edge-touching grains as ½ a grain, $N_{intercepted}$, and corner grains as ¼ a grain. The following Equation (1) determines the number of grains per micrometer squared, $N_{A}$, using magnification, M, and total image area, A:

(1) $$N_{A}=(\frac{M^{2}}{A})(N_{inside}+\frac{1}{2}N_{\mathrm{int}ercepted}+1),$$

The average grain area, $\bar{A}$, is the reciprocal of $N_{A}$. Each area is approximated with diameter, d, as the square root of $\bar{A}$. Using a rectangular area allows for a higher grain count and minimized scatter, which increases accuracy compared to a circular test area. This is especially true in the micrographs with larger grains, such as the ones encompassed in the test dataset. The planimetric method also alleviates any impact of un-equiaxed grain orientations, which is minimal in the test dataset.

### 2.6. Grain Size Determination Method

It is important to note that the goal of the present work is not to match or replace the ASTM or ISO standards but rather to develop a robust quantitative grain size characterization methodology that is accurate, quick, and does not require great input from the researcher. Therefore, rather than grain size number, the output of the algorithm described in this work is the mean grain diameter, which is widely used in characterization.

The method proposed here comprises two main macro-operations: Grain boundary amplification and grain size determination. In the former, an image editing software processes surface adjustments in contrast and intensity to ensure grain boundaries are visible. Metadata are also assigned in this step, with a pixel-to-micrometer ratio inserted under the Exif user comment as described before. GitBash is then used to apply the HED algorithm and transform the edited JPG image into a grain boundary prediction map. The original image is conserved for its metadata use in the following step.

In grain size determination, the PNG output file of HED is converted to grayscale and thresholded to enhance the difference between grains and grain boundaries. In this work, the optimum threshold value was experimentally determined to be 252. Likewise, the optimum number of iterations for erosion and dilation were experimentally determined to be seven and eight, respectively, using a disk kernel of radius 1 pixel to create closed contours and thin boundary walls. A 3 X 3 structural element then creates a mask and labels from the regions inside grain boundary contours. Cells touching the border of the image are cleared so that only full inside cells are considered. Finally, the *regionprops* function within the Scikit module in Python is used to calculate the area of each region, which constitutes the main output.

Toward benchmarking the output of this method against both the standards and the ground truth of a material microstructure, it is important to recall that in polycrystalline single-phase materials, the most energy-favorable grain structure is such that the intersection of three grains forms at dihedral angles of 120° [44], and a truncated octahedron represents this equilibrium microstructure in three-dimensional space. This polyhedron is reduced to a normal hexagon honeycomb structure in a two-dimensional plane. Therefore, in this work, the area of each region ($A_{r}$) is thus taken to be that of a hexagon with an inner diameter (d) resulting from the smallest possible inscribed circle in Equation (2) and an outer diameter (*D*) from the largest possible inscribed circle in Equation (3). The average diameter of the grain is the mean of the outer and inner diameters.

(2) $$d=\frac{2}{3}\sqrt{2A_{r}},$$

(3) $$D=2\sqrt{2A_{r}/3\sqrt{3}},$$

The pixel-to-micrometer ratio is retrieved from the metadata to convert pixel units into micrometers. Grain size is reported as a distribution or a mean of the grains detected. No input other than an image and pixel-to-micrometer ratio is needed.

## 3. Results and Discussion

**Figure 3:**
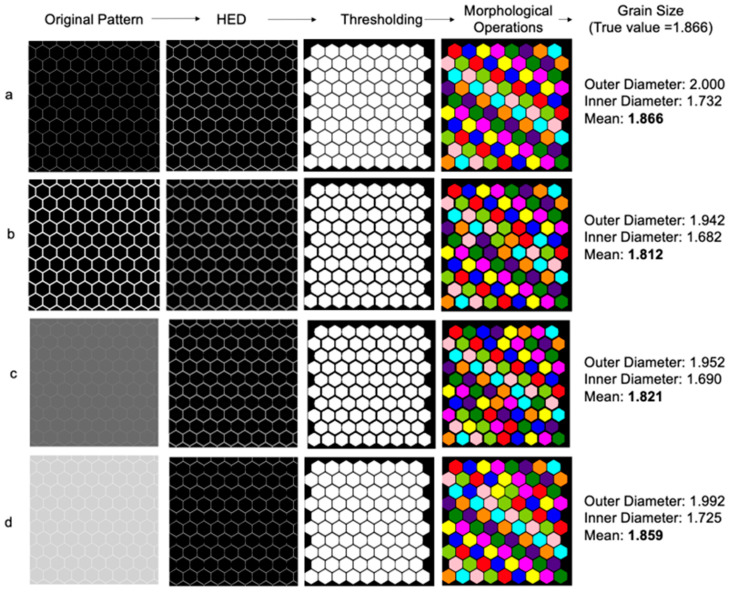
Mean diameter determination of honeycomb structure (**a**) with an expected value of 1.866 units. Alterations of the initial figure include sidewall thickening (**b**), low-intensity low contrast (**c**), and high-intensity low contrast (**d**).

To analyze the effectiveness of the newly proposed grain size determination workflow, a series of tests with increasing complexity were carried out. To begin, a test on a computer-generated honeycomb structure representing an ideal polycrystalline cross-section was executed. The HED, thresholding, morphological operations, and grain size determination processed each image as discussed above (Figure 1c). By using a standard hexagon, the diameter is definitive, with no interpretation or error needed from a standardized method such as Heyn’s or Saltykov’s. The side length of each hexagon was normalized to 1 arbitrary unit, defining the minimum and maximum diameters as 1.732 and 2.000 units, respectively. The resulting mean diameter is 1.866 units. The algorithm reconstructs side lengths from a measured area using Equations (2) and (3). The algorithm results are shown in Figure 3 with images at each processing step. An optimal construct (Figure 3a) and an accurate pixel-to-micrometer ratio result in accurate diameter determination with essentially no error. As expected, thickening the hexagon borders (Figure 3b) decreases grain area and a smaller resulting diameter. The case for underexposed and overexposed micrographs was simulated using Figure 3c,d, respectively. It was found that the HED allows for edge detection at a range of luminosities, although high-intensity, low-contrast images perform marginally better than equivalent images at a low intensity.

Considering that each micrograph has a different associated grain size, normalization is needed for effective comparison. To quantify the size of the error relative to the assumed true value, the percent error formula is used, as shown in Equation (4), which uses a percentage rather than a non-normalized value. In this case, the percent error of the HED workflow is measured in relation to Heyn’s using the algorithm’s calculated diameter, $D_{calc}$, and expected diameter, $D_{exp}$. The most significant deviation from the expected value (Figure 3b) produced a 2.89% error, with a mean overall error for all images of 1.420%.

(4) $$\%\;Error=\left|\frac{D_{calc}-D_{exp}}{D_{exp}}\right|\cdot 100\%\;,$$

The honeycomb test highlights some benefits of this method. Firstly, ignoring edge grains allows for a more accurate representation of the material at hand because only whole grains are considered. Secondly, using Exif metadata to convey a pixel-to-micrometer ratio allows for easy batch operations of similar images. Finally, area approximation accurately represents the mean diameter of a material’s crystalline regions.

The proposed algorithm must also yield accurate results on real micrographs to be considered a viable grain size determination method. Therefore, both new and conventional methods were used to analyze a set of 25 experimental optical microscopy images. Laboratory images contain noise, which poses a specific challenge in disrupting the algorithm’s accuracy in detecting edges. Consequently, to highlight the HED’s response to noise, no external noise reduction functions were used.

Heyn’s method was used as one of the benchmarks with the following basic parameters. All lines were randomly selected before being placed on the image as a mask, but care was taken to ensure all regions of the micrograph were represented. The test lines were oriented so that a wide range of angles was considered. A set of ten lines was found to be sufficient with an average of 127.47 intersections, greater than the 50 minimum recommendation in ASTM E112-13 13.1. The average grain diameter was reported at 61.62 µm with a standard deviation of 5.15 µm. Each micrograph contained approximately 167 whole grains in its field of view (neglecting edge-touching grains).

To determine the robustness of HED in comparison with other edge detection types, the same images were processed with the Canny edge detection method [31]. Thresholding and the number of eroding iterations were adjusted so that contained cells were formed in the Canny method. The rest of the methodology was kept the same. Canny edge detection requires two values of edge thresholding, and 0 and 70 were determined based on visual inspection.

**Figure 4:**
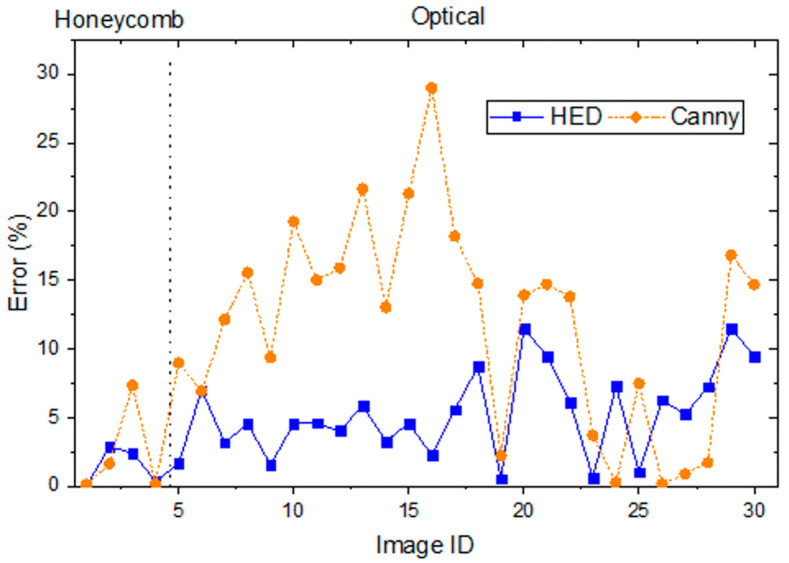
Grain size measured by HED and Canny edge detection algorithms compared to line intercept method reported as % error.

When compared to Heyn’s method, the Canny method exhibited an average error of 11.9% in the optical microscope images tested with a standard deviation of 7.6%. It had an error of 2.3% in hexagon (synthetic) tests (Figure 4). For HED, an average error of 5.1% was obtained for the optical micrographs with a standard deviation of 3.1%. These errors arise from a set of factors for both edge detection techniques:

- *Connected cells*: Low image quality and low-intensity differences around boundaries increase the presence of less-pronounced edges. Weak edges may not be detected, forming a cluster of adjacent cells. Although morphological operands greatly reduce the effect of connected cells, large distances between broken ends cannot be joined unless a significant amount of erosion is used, which, in turn, could erroneously fill smaller legitimate cells; therefore, the number of erosion iterations must be limited. The net result is that the area of a supercell is taken as that of a single cell, which results in a larger average grain size.
- *Surface defects*: Edge detection does not discern between grain boundaries and blemishes. Surface defects such as rough topological surfaces and loose powder have apparent edges, which edge detection methods perceive as grain boundaries. When a substantial portion of a surface defect is detected relative to the size of the grains around it, the algorithms process the surface defect as an individual cell or as part of a grain boundary.

The lower error in HED indicates better handling of these issues, particularly connected cells, although the 5.1% error is still rather large. It has to be stressed that this error is with respect to Heyn’s method output, which is not necessarily the ground truth but rather the agreed-upon ASTM technical standard.

**Figure 5:**
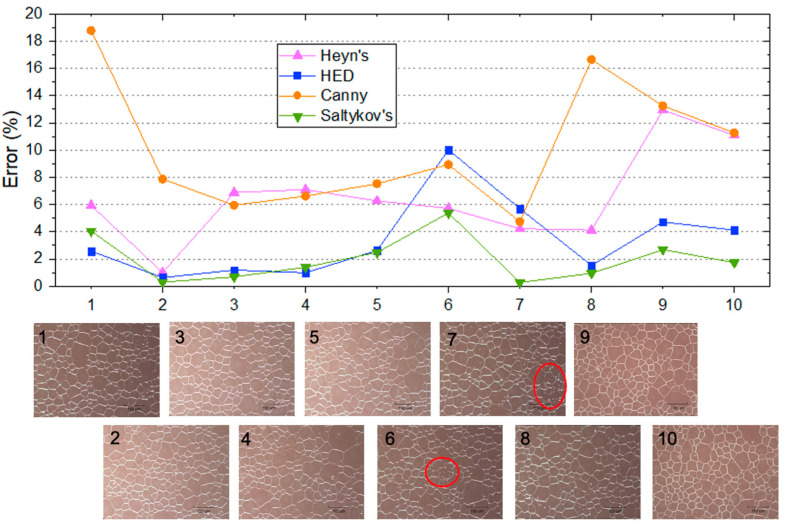
A comparison of four grain determination methods’ error to the average grain Haywood diameter (ground truth). For reference, the micrographs are located underneath, with areas of lower resolution circled in red.

Therefore, considering that all grain size determination methods inevitably possess approximation errors, a comparison with a more accurate benchmark is needed. Consequently, to obtain the average ground truth diameter, each cell’s Heywood diameter [45] is taken. A closed contour is drawn manually using ImageJ software around each interior grain. The area is calculated and equated to the area of a circle with a constant diameter, *d_H_*. The average of all Heywood diameters is taken to represent the true average diameter of the micrograph. A subset of 10 micrographs from the original 25 was selected and measured using Heyn’s, Saltykov’s, Canny edge detection, and HED methods (Figure 5).

The HED method had a lower error than Heyn’s and Canny edge detection for 8 of the 10 micrographs with an average of 3.4% compared to 6.5% (Heyn’s) and 10.2% (Canny). The two micrographs where this was not the case are cases in which a portion of the image is not fully resolved due to blurriness and unclear grain boundaries (Figure 5). In these regions, it is challenging to determine grain boundaries even in the visual analysis, as they are not well defined. Saltykov’s method (planimetric) reliably had the most accurate grain size, except for two images (1 and 4) where HED slightly outperformed it.

It is important to note the accuracy of Heyn’s method is dependent on the number of intersected grain boundaries (ASTM E112-13 13.1), meaning the error of Heyn’s is not set at the values obtained. Increasing the number of test lines and intersections would undoubtedly increase Heyn’s accuracy albeit at the cost of measurement time.

**Figure 6:**
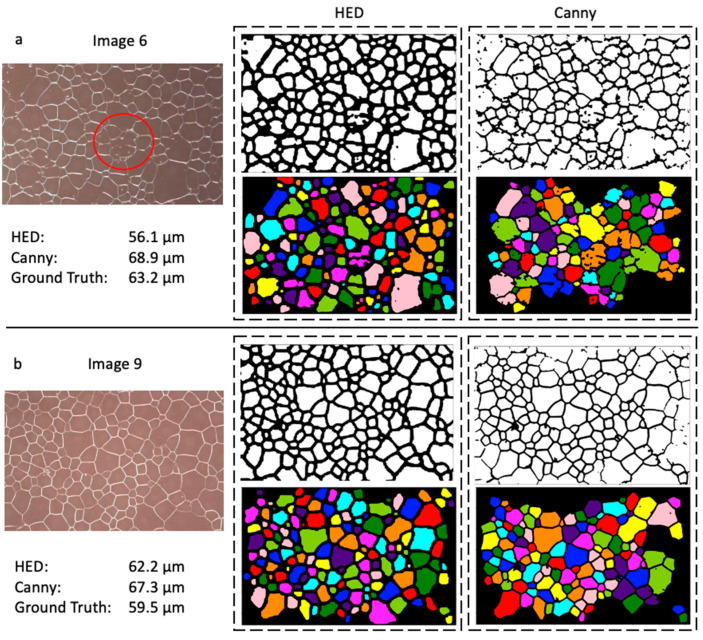
A visual representation of HED and Canny processing methods after morphological operations on image 6 (**a**) and image 9 (**b**) from Figure 5. The circled area represents a zone of low resolution.

A qualitative analysis of the output images sheds further light on the methods’ respective quantification of grain size and error rates. The edge detections’ outputs from a generally noiseless micrograph are seen in Figure 6b. One can observe the thick, encapsulating borders that characterize the HED’s thresholded image. Comparatively, Canny’s edge detection outputs thinner and less well-connected edges, resulting in numerous connected cells towards the right and top of the image, hence the larger black space in the same regions. HED outperformed Canny with an error rate of 4.7% to Canny’s 13.2% for this micrograph. Generally, however, very clear images are similarly processed by each algorithm. It becomes apparent that HED is particularly superior when poorer-defined grain boundaries and noise are introduced. Despite having a higher error in image 6 (Figure 6a), the HED results demonstrate many less-connected cells. Artifacts near the edges of optical images are common [13] and seem to have minimal impact on the HED workflow. Meanwhile, large portions of near-edge and interior grains are not properly contoured with Canny edge detection, which artificially increased its calculated grain size. Both algorithms displayed difficulty in the poorly resolved region of the micrograph (circled in red in Figure 6a), indicating proper imaging techniques are essential in any automated grain size determination method.

The time cost of each method was analyzed using an Intel Xeon E5-1603 CPU at 2.8 GHz (Table 1) and an Nvidia GeForce GTX 1080 GPU with 8 GB of RAM. All methods were tested on the images in Figure 5. It is worth noting that the elapsed time needed to carry out Heyn’s, Saltykov’s, and Heywood’s mean diameter is sensitive to the micrograph characteristics and measurement parameters: More grains and test lines will cause significantly longer measuring times. The elapsed time also depends on the researcher’s skill and familiarity with the analysis. Saltykov’s method’s high accuracy compared to the measured Heywood diameters (Figure 5) is associated with a high yield time (Table 1). By contrast, edge detection techniques, however, are bound by computational capabilities and the size of the image. For example, when the methods were run using an Nvidia A100, the computing time for HED was reduced from 6.4 ± 0.3 s to 5.6 ± 0.5 s. Nonetheless, it is clear that automatic methods are faster irrespective of the image type. Although Canny edge detection had the shortest computing time (Table 1), it also showed the highest error against Heywood’s ground truth average diameter (Figure 5).

The time taken to compute the edge detection techniques does not include the time taken to determine the pixel-to-micrometer ratio and cropping as such is performed in batches in an external application. Preprocessing normally took less than 2 min to complete for 25 images. The time to execute the HED method using the GeForce 1080 on one image is split between HED in GitBash- 6.4 s and grain size determination in a Jupyter notebook- 0.66 s. A 25-image batch took 3 min and 6 s to complete an average time of 6.7 s per image. A large portion of that time (2 min and 48 s) was taken by HED. The same batch operation using the A100 GPU took 2 min and 33 s—an average time of 6.1 s per image. Canny edge detection is a native attribute to Python cv2; therefore, its time is entirely measured in a Jupyter notebook. It is worth mentioning that the Jupyter notebooks used in HED and Canny methods were restarted between each image ticket to time the process per image.

The suggested image processing workflow is a faster alternative to conventional methods. Batch processing allows for the measurement of multiple images, with each micrograph taking a fraction of the time manual measurement requires. Moreover, the skill and knowledge requisites of the researcher are significantly reduced. In addition, operator error, for example in counting grains or grain intersections, is eliminated in an automated process. No manual intervention is required once the image is input. In essence, the responsibilities of the researcher are reduced to cropping and determining the number of pixels per unit length. Whenever faulty results are obtained, filtering to accentuate grain boundaries can be performed before the input.

The present workflow has been shown to measure the grain diameter in optical micrographs accurately and fast. It is important to recall that for grain size determination, it is beneficial to image multiple sections of material to ensure that a representative sample of the microstructure is being analyzed. Typically, this process has been time consuming and thus grain size measurements are typically performed on just one image. The current workflow can overcome this limitation.

Moreover, considering the method comparison presented in Figure 4, it is clear that the HED method has a lower error than Heyn’s method for most samples; as such, if the ten randomly placed lines in Heyn’s are taken as a sufficiently accurate measurement of the mean grain diameter, the HED method can certainly be considered a viable and preferred alternative.

The purpose of the HED workflow is to underscore the applicability of pre-trained CNNs to the characterization of micrographs. The lack of retraining avoids possible overfitting and adequate expertise and resources needed for modifying a CNN. Currently, the algorithm has been determined to be accurate for determining the grain size in optical micrographs possessing well-defined grain boundaries; however, the universality of the algorithm, unbounded by training to a specific dataset, does not restrict uses to the ceria proof of concept material used in this work. Relatively large grains are also needed, as aggregates of small contours are either not detected by HED or filled in morphological erosion. However, experimentally, micrographs (optical or electron) are collected at magnifications that capture multiple grains with sufficient grain boundary detail. In essence, an adequate publication- or research-quality material micrograph is expected to be correctly measured using this HED workflow.

It is nonetheless clear that future work should test the robustness of the algorithm with a larger, more diverse dataset and compare it to conventional workflows. Thinning and seeding techniques seen in past research [12] may also improve results by removing spurious edges. Filtering and other processing may be required to expand the applications to SEM micrographs.

Further application of the grain size determination workflow is encouraged, especially in micrographs containing similar characteristics to those in this work. Similar techniques may also be employed in other materials’ science image segmentation domains where feature size is relevant.

## 4. Conclusions

Grain size is an essential statistic in the characterization of materials. Manual grain size determination is tedious and lengthy, but its automation is a complex process. Key issues such as image quality and noise pose difficulty for even robust algorithms. In the presented study, the average grain diameters of microstructure images were quantified by the use of a novel pre-trained model and post-processing. The HED method demonstrated superior recognition of grain boundaries compared to Canny edge detection. The workflow can sufficiently quantify the mean diameter in micrographs with distinct and closed grain boundaries. The low error obtained compared to conventional methods demonstrates the potential of using pretrained CNNs for automated material characterization.

## Figures and Tables

**Table 1:** Time requirements of different grain diameter analyses of one micrograph, in seconds. The Heywood diameter is set as the ground truth value and thus does not have an associated error.

Category	Heyn	HED *	Canny	Saltykov	Heywood
Average Time (s) **	217 ± 20	9.90 ± 0.5	4.25 ± 0.1	195 ± 10	916 ± 63
Manual Input	Y	N	N	Y	Y
Error to Ground Truth ***	6.5%	3.4%	10.2%	2.0%	-

* Using A100 GPU, ** ± reported in standard deviation, *** Heywood diameter.

## Data Availability

All data and code are available on GitHub and can be made available upon request by contacting the corresponding authors.

## Sample Availability

Samples of the compounds are available from the authors upon reasonable request.
